# SrMn^II^_2_Mn^III^(PO_4_)_3_

**DOI:** 10.1107/S1600536813020977

**Published:** 2013-08-14

**Authors:** Ghaleb Alhakmi, Abderrazzak Assani, Mohamed Saadi, Claudine Follet, Lahcen El Ammari

**Affiliations:** a Laboratoire de Chimie du Solide Appliquée, Faculté des Sciences, Université Mohammed V-Agdal, Avenue Ibn Battouta, BP 1014, Rabat, Morocco; b Laboratoire des Matériaux Céramiques et Procédés Associés EA 2443, Université de Valenciennes et du Hainaut – Cambrésis Le Mont Houy, 59313 Valenciennes, France

## Abstract

The title compound, strontium trimanganese tris­(ortho­phosphate), was synthesized under hydro­thermal conditions. Its structure is isotypic to that of the lead analogue PbMn^II^_2_Mn^III^(PO_4_)_3_. Two O atoms are in general positions, whereas all others atoms are in special positions. The Sr and one P atom exhibit *mm*2 symmetry, the Mn^II^ atom 2/*m* symmetry, the Mn^III^ atom and the other P atom .2. symmetry and two O atoms are located on mirror planes. The three-dimensional network of the crystal structure is made up of two types of chains running parallel to [010]. One chain is linear and is composed of alternating Mn^III^O_6_ octa­hedra and PO_4_ tetra­hedra sharing vertices; the other chain has a zigzag arrangement and is built up from two edge-sharing Mn^II^O_6_ octa­hedra connected to PO_4_ tetra­hedra by edges and vertices. The two types of chains are linked through PO_4_ tetra­hedra, leading to the formation of channels parallel to [100] and [010] in which the Sr^II^ ions are located. They are surrounded by eight O atoms in the form of a slightly distorted bicapped trigonal prism.

## Related literature

For the isotypic lead analogue, see: Alhakmi *et al.* (2013 ▶). For compounds with related structures, see: Adam *et al.* (2009 ▶); Assani *et al.* (2011*a* ▶,*b* ▶,*c* ▶); Moore & Ito (1979 ▶). For applications of related compounds, see: Trad *et al.* (2010 ▶). For the by-product phase, see: Moore & Araki (1973 ▶). For bond-valence analysis, see: Brown & Altermatt (1985 ▶).

## Experimental

### 

#### Crystal data

SrMn_3_(PO_4_)_3_
*M* *_r_* = 537.35
Orthorhombic, 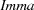

*a* = 10.2373 (10) Å
*b* = 13.8981 (15) Å
*c* = 6.6230 (6) Å
*V* = 942.31 (16) Å^3^
*Z* = 4
Mo *K*α radiation
μ = 10.14 mm^−1^
*T* = 296 K
0.28 × 0.15 × 0.12 mm

#### Data collection

Bruker APEXII diffractometer
Absorption correction: multi-scan (*SADABS*; Bruker, 2009 ▶) *T*_min_ = 0.164, *T*_max_ = 0.376
4726 measured reflections
991 independent reflections
877 reflections with *I* > 2σ(*I*)
*R*_int_ = 0.040

#### Refinement

*R*[*F* ^2^ > 2σ(*F* ^2^)] = 0.025
*wR*(*F* ^2^) = 0.066
*S* = 1.04
991 reflections
53 parameters
Δρ_max_ = 0.83 e Å^−3^
Δρ_min_ = −0.92 e Å^−3^

### 

Data collection: *APEX2* (Bruker, 2009 ▶); cell refinement: *SAINT* (Bruker, 2009 ▶); data reduction: *SAINT*; program(s) used to solve structure: *SHELXS97* (Sheldrick, 2008 ▶); program(s) used to refine structure: *SHELXL97* (Sheldrick, 2008 ▶); molecular graphics: *ORTEP-3 for Windows* (Farrugia, 2012 ▶) and *DIAMOND* (Brandenburg, 2006 ▶); software used to prepare material for publication: *publCIF* (Westrip, 2010 ▶).

## Supplementary Material

Crystal structure: contains datablock(s) I. DOI: 10.1107/S1600536813020977/wm2761sup1.cif

Structure factors: contains datablock(s) I. DOI: 10.1107/S1600536813020977/wm2761Isup2.hkl

Additional supplementary materials:  crystallographic information; 3D view; checkCIF report

## Figures and Tables

**Fig. 1.**
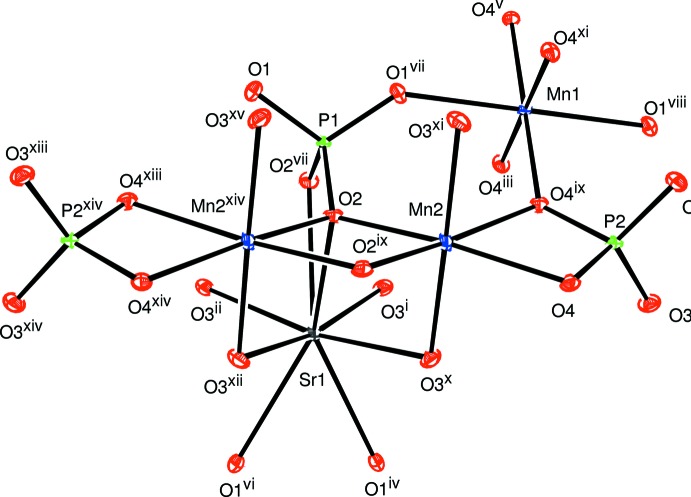
The main building units of the crystal structure of the title compound. Displacement ellipsoids are drawn at the 50% probability level. [Symmetry codes: (i) x, -y + 1, -z; (ii) -x, y - 1/2, -z; (iii) x, y - 1/2, -z; (iv) -x, -y + 1, -z; (v) x, y, z - 1; (vi) -x, -y + 1/2, z - 1; (vii) -x, -y + 1/2, z; (viii) -x + 1/2, y - 1/2, z + 1/2; (ix) x - 1/2, -y + 1/2, -z + 1/2; (x) -x + 1/2, -y + 1/2, -z + 1/2; (xi) x - 1/2, y - 1/2, z + 1/2; (xii) -x, -y, -z + 1; (xiii) -x + 1/2, -y + 1, z + 1/2; (xiv) -x + 1/2, y, -z + 1/2.]

**Fig. 2.**
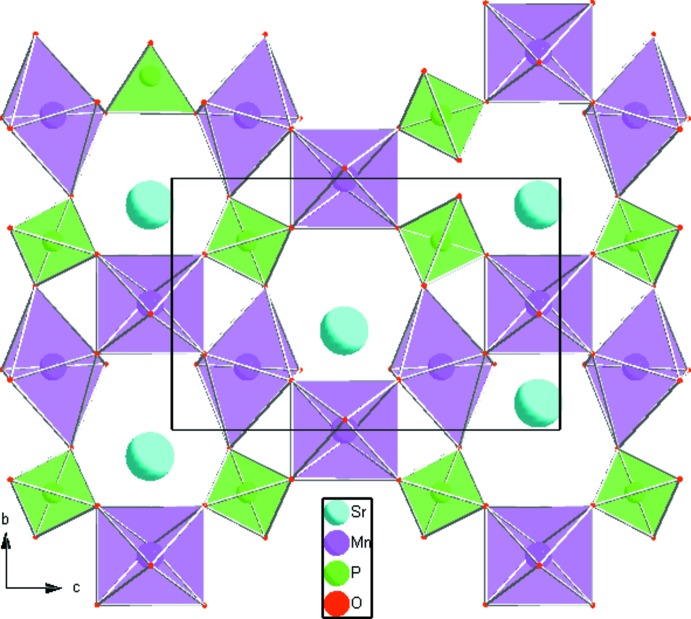
Polyhedral representation of SrMn3(PO4)3 with channels running parallel to [100].

## supplementary crystallographic information

### 1. Comment

Hydrothermal studies in the systems *A*_2_O–*M*O–P_2_O_5_ and *M*'O–*M*O–P_2_O_5_ led to new phosphates with framework structures closely related to that of the alluaudite [(Na,Ca)Mn^II^(Fe^III^,Mn^III^,Fe^II^Mg)_2_(PO_4_)_3_] structure type. For instance, the phosphates Ag_2_*M*_3_(HPO_4_)(PO_4_)_2_ (*M* = Co, Ni) (Assani *et al.*, 2011**a*,b* and PbMn^II^_2_Mn^III^(PO_4_)_3_ (Alhakmi *et al.*, 2013) were prepared this way. Their compositions can be represented by the general formula (*A*1)(*A*2)(*M*1)(*M*2)_2_(PO_4_)_3_ as introduced by Moore & Ito (1979) for alluaudite-related compounds. In this nomenclature the order of the cations *A* and *M* is related to the decreasing size of the discrete sites. Mainly, the *A* sites can be occupied by either mono- or divalent medium-sized cations while the *M* cationic sites correspond to an octahedral environment generally occupied by transition metal cations. In the case of alluaudite-type compounds, a great field of applications such as positive electrodes in lithium and sodium batteries (Trad *et al.*, 2010) has been established. Our focus of investigation is associated with mixed-cations orthophosphates that are related to the above mentioned compounds. By means of the hydrothermal synthesis method, we have recently prepared and structurally characterized (*A*1)(*A*2)(*M*1)(*M*2)_2_(PO_4_)_3_ phosphates (Assani *et al.*, 2011*c*). The present paper describes the synthesis and structural characterization of the mixed-valent Mn^II,III^ phosphate with composition SrMn^II^_2_Mn^III^(PO_4_)_3_. Such Mn^II,III^ systems are rather scarce (Adam *et al.*, 2009). The structure of the title compound is isotypic to that of the lead analogue PbMn^II^_2_Mn^III^(PO_4_)_3_ (Alhakmi *et al.*, 2013)

Except two oxygen atoms (O3, O4) in general positions, all other atoms are located on special positions of space group *Imma*. The connection of the metal-oxygen polyhedra, *viz*. SrO_8_ polyhedra, MnO_6_ octahedra and PO_4_ tetrahedra is shown in Fig. 1. The crystal structure consists of two isolated PO_4_ tetrahedra linked to two types of MnO_6_ octahedra, building two different chains running parallel to [010]. The first chain is formed by alternating Mn^III^O_6_ octahedra and PO_4_ tetrahedra by sharing vertices. The second chain is built up from two adjacent edge-sharing octahedra (Mn^II^_2_O_10_ dimers) that are further linked to two PO_4_ tetrahedra by a common edge. These two types of chains are linked together by common vertices of PO_4_ tetrahedra to form a porous three-dimensional framework that delimits two types of tunnels parallel to [100] and [010] where the Sr^II^ ions are located (Fig. 2). The coordination sphere of the alkaline earth metal ions is that of a bicapped trigonal prism.

Bond valence calculations (Brown & Altermatt, 1985) of SrMn^II^_2_Mn^III^(PO_4_)_3_ revealed bond valence sums for Sr1^II+^, Mn1^III+^, Mn2^II+^, P1^V+^ and P2^V+^ that are close to the expected values, *viz*. 1.81, 3.06, 1.98, 5.02 and 4.87 valence units (v.u.), respectively. The bond valence sums calculated for all O atoms are in the range of 1.79 – 2.04 v.u., thus confirming the validity of the structure model.

The framework of the title compound shows some resemblance to that of Ag_2_*M*_3_(HPO_4_)(PO_4_)_2_ phosphates (*M* = Ni, Co; Assani *et al.*, 2011**a*,b*), whereby the two Ag^I^ cations in the channels are replaced by Sr^II^.

### 2. Experimental

Crystals of the title compound were isolated from the hydrothermal treatment of a reaction mixture of strontium, manganese and phosphate precursors in a proportion corresponding to the molar ratio Sr:Mn:P = 1: 3: 3. The hydrothermal reaction was conducted in a 23 ml Teflon-lined autoclave, filled to 50% with distilled water and under autogeneous pressure at 478 K for five days. After being filtered off, washed with deionized water and air dried, the reaction product consisted of brown sheet-shaped crystals corresponding to the title compound. Besides, parallelepipedic colourless crystals were present which were identified to be Mn_5_(HPO_4_)_2_(PO_4_)_2_^.^4H_2_O (Moore & Araki, 1973).

### 3. Refinement

The highest and lowest remaining electron density peaks in the final Fourier map are 0.71 Å and 0.49 Å, respectively, away from O3 and P2.

### Crystal data

**Table:**

SrMn_3_(PO_4_)_3_	*F*(000) = 1016
*M**_r_* = 537.35	*D*_x_ = 3.788 Mg m^−^^3^
Orthorhombic, *I**m**m**a*	Mo *K*α radiation, λ = 0.71073 Å
Hall symbol: -I 2b 2	Cell parameters from 991 reflections
*a* = 10.2373 (10) Å	θ = 2.9–33.3°
*b* = 13.8981 (15) Å	µ = 10.14 mm^−^^1^
*c* = 6.6230 (6) Å	*T* = 296 K
*V* = 942.31 (16) Å^3^	Sheet, brown
*Z* = 4	0.28 × 0.15 × 0.12 mm

### Data collection

**Table:**

Bruker APEXII diffractometer	991 independent reflections
Radiation source: fine-focus sealed tube	877 reflections with *I* > 2σ(*I*)
Graphite monochromator	*R*_int_ = 0.040
φ and ω scans	θ_max_ = 33.3°, θ_min_ = 2.9°
Absorption correction: multi-scan (*SADABS*; Bruker, 2009)	*h* = −15→15
*T*_min_ = 0.164, *T*_max_ = 0.376	*k* = −21→10
4726 measured reflections	*l* = −9→10

### Refinement

**Table:**

Refinement on *F*^2^	0 restraints
Least-squares matrix: full	Primary atom site location: structure-invariant direct methods
*R*[*F*^2^ > 2σ(*F*^2^)] = 0.025	Secondary atom site location: difference Fourier map
*wR*(*F*^2^) = 0.066	*w* = 1/[σ^2^(*F*_o_^2^) + (0.0389*P*)^2^] where *P* = (*F*_o_^2^ + 2*F*_c_^2^)/3
*S* = 1.04	(Δ/σ)_max_ < 0.001
991 reflections	Δρ_max_ = 0.83 e Å^−^^3^
53 parameters	Δρ_min_ = −0.92 e Å^−^^3^

### Special details

**Table:**

Geometry. All s.u.'s (except the s.u. in the dihedral angle between two l.s. planes) are estimated using the full covariance matrix. The cell s.u.'s are taken into account individually in the estimation of s.u.'s in distances, angles and torsion angles; correlations between s.u.'s in cell parameters are only used when they are defined by crystal symmetry. An approximate (isotropic) treatment of cell s.u.'s is used for estimating s.u.'s involving l.s. planes.
Refinement. Refinement of *F*^2^ against all reflections. The weighted *R*-factor *wR* and goodness of fit *S* are based on *F*^2^, conventional *R*-factors *R* are based on *F*, with *F* set to zero for negative *F*^2^. The threshold expression of *F*^2^ > σ(*F*^2^) is used only for calculating *R*-factors(gt) *etc*. and is not relevant to the choice of reflections for refinement. *R*-factors based on *F*^2^ are statistically about twice as large as those based on *F*, and *R*- factors based on all data will be even larger.

### Fractional atomic coordinates and isotropic or equivalent isotropic displacement parameters (Å^2^)

**Table:**

	*x*	*y*	*z*	*U*_iso_*/*U*_eq_	
Sr1	0.0000	0.2500	−0.10600 (6)	0.00886 (11)	
Mn1	0.0000	0.5000	0.5000	0.00446 (14)	
Mn2	0.2500	0.36768 (4)	0.2500	0.00742 (12)	
P1	0.0000	0.2500	0.40707 (15)	0.00402 (19)	
P2	0.2500	0.57346 (6)	0.2500	0.00591 (16)	
O1	0.0000	0.16039 (16)	0.5390 (3)	0.0089 (4)	
O2	0.1174 (2)	0.2500	0.2602 (3)	0.0080 (4)	
O3	0.20437 (17)	0.63359 (12)	0.0726 (2)	0.0101 (3)	
O4	0.36220 (15)	0.50005 (12)	0.1971 (2)	0.0079 (3)	

### Atomic displacement parameters (Å^2^)

**Table:**

	*U*^11^	*U*^22^	*U*^33^	*U*^12^	*U*^13^	*U*^23^
Sr1	0.01068 (19)	0.0107 (2)	0.00518 (18)	0.000	0.000	0.000
Mn1	0.0042 (3)	0.0064 (3)	0.0027 (3)	0.000	0.000	0.0000 (2)
Mn2	0.0095 (2)	0.0054 (2)	0.0073 (2)	0.000	−0.00037 (15)	0.000
P1	0.0052 (4)	0.0042 (5)	0.0027 (4)	0.000	0.000	0.000
P2	0.0072 (3)	0.0062 (4)	0.0043 (3)	0.000	0.0006 (2)	0.000
O1	0.0111 (10)	0.0067 (10)	0.0089 (9)	0.000	0.000	0.0021 (8)
O2	0.0075 (10)	0.0099 (10)	0.0065 (10)	0.000	0.0031 (7)	0.000
O3	0.0126 (7)	0.0102 (8)	0.0074 (7)	0.0023 (6)	0.0006 (6)	0.0026 (6)
O4	0.0087 (7)	0.0079 (7)	0.0072 (7)	0.0004 (6)	0.0033 (5)	0.0006 (5)

### Geometric parameters (Å, º)

**Table:**

Sr1—O3^i^	2.6540 (17)	Mn2—O2	2.1265 (15)
Sr1—O3^ii^	2.6540 (17)	Mn2—O2^xiii^	2.1265 (15)
Sr1—O3^iii^	2.6540 (17)	Mn2—O3^i^	2.1869 (16)
Sr1—O3^iv^	2.6540 (17)	Mn2—O3^ix^	2.1869 (16)
Sr1—O1^v^	2.660 (2)	Mn2—O4	2.1969 (17)
Sr1—O1^vi^	2.660 (2)	Mn2—O4^xi^	2.1969 (17)
Sr1—O2	2.707 (2)	P1—O1^vii^	1.522 (2)
Sr1—O2^vii^	2.707 (2)	P1—O1	1.522 (2)
Mn1—O4^viii^	1.9219 (15)	P1—O2^vii^	1.546 (2)
Mn1—O4^ix^	1.9219 (15)	P1—O2	1.546 (2)
Mn1—O4^x^	1.9219 (15)	P2—O3^xi^	1.5158 (16)
Mn1—O4^xi^	1.9219 (15)	P2—O3	1.5158 (16)
Mn1—O1^xii^	2.244 (2)	P2—O4^xi^	1.5758 (17)
Mn1—O1^vii^	2.244 (2)	P2—O4	1.5758 (17)
			
O3^i^—Sr1—O3^ii^	75.12 (8)	O4^ix^—Mn1—O1^xii^	94.51 (6)
O3^i^—Sr1—O3^iii^	170.43 (7)	O4^x^—Mn1—O1^xii^	94.51 (6)
O3^ii^—Sr1—O3^iii^	104.06 (8)	O4^xi^—Mn1—O1^xii^	85.49 (6)
O3^i^—Sr1—O3^iv^	104.06 (8)	O4^viii^—Mn1—O1^vii^	94.51 (6)
O3^ii^—Sr1—O3^iv^	170.43 (7)	O4^ix^—Mn1—O1^vii^	85.49 (6)
O3^iii^—Sr1—O3^iv^	75.12 (8)	O4^x^—Mn1—O1^vii^	85.49 (6)
O3^i^—Sr1—O1^v^	111.04 (5)	O4^xi^—Mn1—O1^vii^	94.51 (6)
O3^ii^—Sr1—O1^v^	77.78 (4)	O1^xii^—Mn1—O1^vii^	180.0
O3^iii^—Sr1—O1^v^	77.78 (4)	O2—Mn2—O2^xiii^	79.45 (9)
O3^iv^—Sr1—O1^v^	111.04 (5)	O2—Mn2—O3^i^	83.60 (7)
O3^i^—Sr1—O1^vi^	77.78 (4)	O2^xiii^—Mn2—O3^i^	95.69 (7)
O3^ii^—Sr1—O1^vi^	111.04 (5)	O2—Mn2—O3^ix^	95.69 (7)
O3^iii^—Sr1—O1^vi^	111.04 (5)	O2^xiii^—Mn2—O3^ix^	83.60 (7)
O3^iv^—Sr1—O1^vi^	77.78 (4)	O3^i^—Mn2—O3^ix^	179.07 (9)
O1^v^—Sr1—O1^vi^	55.83 (10)	O2—Mn2—O4	169.37 (7)
O3^i^—Sr1—O2	64.86 (5)	O2^xiii^—Mn2—O4	107.77 (6)
O3^ii^—Sr1—O2	64.86 (5)	O3^i^—Mn2—O4	87.85 (6)
O3^iii^—Sr1—O2	105.98 (5)	O3^ix^—Mn2—O4	92.92 (6)
O3^iv^—Sr1—O2	105.98 (5)	O2—Mn2—O4^xi^	107.77 (6)
O1^v^—Sr1—O2	142.35 (4)	O2^xiii^—Mn2—O4^xi^	169.37 (7)
O1^vi^—Sr1—O2	142.35 (4)	O3^i^—Mn2—O4^xi^	92.92 (6)
O3^i^—Sr1—O2^vii^	105.98 (5)	O3^ix^—Mn2—O4^xi^	87.85 (6)
O3^ii^—Sr1—O2^vii^	105.98 (5)	O4—Mn2—O4^xi^	66.27 (8)
O3^iii^—Sr1—O2^vii^	64.86 (5)	O1^vii^—P1—O1	109.88 (18)
O3^iv^—Sr1—O2^vii^	64.86 (5)	O1^vii^—P1—O2^vii^	111.19 (6)
O1^v^—Sr1—O2^vii^	142.35 (4)	O1—P1—O2^vii^	111.19 (6)
O1^vi^—Sr1—O2^vii^	142.35 (4)	O1^vii^—P1—O2	111.19 (6)
O2—Sr1—O2^vii^	52.72 (9)	O1—P1—O2	111.19 (6)
O4^viii^—Mn1—O4^ix^	180.0	O2^vii^—P1—O2	102.03 (17)
O4^viii^—Mn1—O4^x^	85.55 (10)	O3^xi^—P2—O3	113.08 (14)
O4^ix^—Mn1—O4^x^	94.45 (10)	O3^xi^—P2—O4^xi^	114.15 (9)
O4^viii^—Mn1—O4^xi^	94.45 (10)	O3—P2—O4^xi^	107.75 (9)
O4^ix^—Mn1—O4^xi^	85.55 (10)	O3^xi^—P2—O4	107.75 (9)
O4^x^—Mn1—O4^xi^	180.0	O3—P2—O4	114.15 (9)
O4^viii^—Mn1—O1^xii^	85.49 (6)	O4^xi^—P2—O4	99.29 (12)

Symmetry codes: (i) *x*, −*y*+1, −*z*; (ii) *x*, *y*−1/2, −*z*; (iii) −*x*, *y*−1/2, −*z*; (iv) −*x*, −*y*+1, −*z*; (v) *x*, *y*, *z*−1; (vi) −*x*, −*y*+1/2, *z*−1; (vii) −*x*, −*y*+1/2, *z*; (viii) *x*−1/2, *y*, −*z*+1/2; (ix) −*x*+1/2, −*y*+1, *z*+1/2; (x) *x*−1/2, −*y*+1, *z*+1/2; (xi) −*x*+1/2, *y*, −*z*+1/2; (xii) *x*, *y*+1/2, −*z*+1; (xiii) −*x*+1/2, −*y*+1/2, −*z*+1/2.
